# Health-related quality of life and mental health in children and adolescents with strabismus – results of the representative population-based survey KiGGS

**DOI:** 10.1186/s12955-019-1144-7

**Published:** 2019-05-07

**Authors:** Alexander K. Schuster, Heike M. Elflein, Roman Pokora, Martin Schlaud, Franz Baumgarten, Michael S. Urschitz

**Affiliations:** 1 Department of Ophthalmology, University Medical Center of the Johannes Gutenberg-University, Mainz, Germany; 2 Division of Pediatric Epidemiology, Institute of Medical Biostatistics, Epidemiology and Informatics, University Medical Center of the Johannes Gutenberg-University, Mainz, Germany; 3 Department of Epidemiology and Health Monitoring, Robert Koch Institute, Berlin, Germany

**Keywords:** Epidemiology, Strabismus, Children, Adolescents, Quality of life, Mental health

## Abstract

**Background:**

To estimate the effect of strabismus (squinting) on mental health and health-related quality of life aspects in children and adolescents.

**Methods:**

Data from the German Health Interview and Examination Survey for Children and Adolescents KiGGS (2003–2006 baseline survey; *N* = 14,835, aged 3 to 17 years, 49% girls) were examined. The presence of strabismus was derived by parental questionnaire, and health-related quality of life and mental health were investigated with the KINDL-R and Strengths and Difficulties Questionnaire. Associations between strabismus and outcomes were analyzed using multivariable linear and logistic regression models.

**Results:**

Of 12,989 children without missing data, 579 children (4.5% of the sample) were reported to have strabismus. Children with strabismus had lower scores in the parent-reported KINDL-R total scale (adjusted beta = − 1.02; 95%CI: -1.86 to − 0.18; *p* = 0.018) and sub-scale ‘friends’ (adjusted beta = − 2.18; 95%CI: -3.56 to -0.80; *p* = 0.002) compared to children without strabismus. The presence of strabismus was also associated with more mental health problems like ‘hyperactivity/inattention’ (adjusted OR = 1.50; 95%CI: 1.14 to 1.98; *p* = 0.005), and ‘peer problems’ (adjusted OR = 1.35; 95%-CI: 1.05 to 1.74; p = 0.018) as reported by parents.

**Conclusions:**

Strabismus in children and adolescents is associated with lower health-related quality of life.

**Electronic supplementary material:**

The online version of this article (10.1186/s12955-019-1144-7) contains supplementary material, which is available to authorized users.

## Background

People with strabismus (squinting) may confuse other people due to the deviating position of one eye [1, 2], and the vis-à-vis may not be able to determine which eye is fixating. Moreover, people with strabismus often do not look others in the eye.

The most common causes of strabismus are infantile esotropia, accommodative esotropia, and other deviations that may be linked with other disorders of the brain.

Manifestation of strabismus may lead to lower self-esteem [3] and potentially impacts activities of daily life and social participation. For example, children with strabismus are less likely to be invited to their peers’ birthday parties compared to those without strabismus [4]. A recent population-based study in the U.S. reported that general health-related quality of life is lower in preschool children having strabismus compared to children without strabismus [5]. To overcome the miss-alignment of both eyes, strabismus surgery can be performed. Nevertheless, the re-alignment does not lead to stereopsis in most cases, but rather reconstructs the face appearance.

Beyond health-related quality of life, chronic health conditions such as strabismus may have negative impacts on the psycho-social wellbeing and development of children. It has been reported that mental health impairments like anxiety and depression are highly prevalent among children with strabismus [6]. However, it remains unclear whether these findings are independent of age, socio-economic status, and other important confounders and whether these associations can be replicated in samples of European children and adolescents.

We therefore aimed to investigate the association of strabismus and various aspects of mental health and health-related quality of life in a large representative population-based cross-sectional sample of children and adolescents living in Germany.

## Methods

### Study design

The German Health Interview and Examination Survey for Children and Adolescents KiGGS is an ongoing repetitive cross-sectional study conducted by the Robert Koch Institute [7]. The aim of KiGGS is to collect the health data of children and adolescents aged between 0 and 17 years. In the baseline survey performed between 2003 and 2006, 17,640 children and adolescents from 167 German cities and municipalities were investigated. A random sample of children and adolescents were chosen based on local population registry data. Parents and their children were contacted and invited to participate. Four study teams covered the 167 study locations within 3 years. The questionnaires were distributed and completed in local study centers. The resulting study sample was representative of the resident population in Germany aged 0–17 years. To ensure a high degree of standardization, the survey teams were trained at the outset of the study and as well as on an on-going basis. Quality management included internal and independent external quality control of data collection and data processing. The KiGGS study was approved by the Charité/Universitätsmedizin Berlin ethics committee and the Federal Office for the Protection of Data in Germany. For the present analysis, the Public Use File “KiGGS 2003–2006” [8] was used.

### Measures

#### Definition of strabismus

In the KiGGS 2003–2006 baseline survey, past or present occurrence of strabismus was obtained by the question: “Has your child ever had a visual dysfunction?” (in German: “Hatte Ihr Kind jemals eine Fehlsichtigkeit?”) with possible answers ‘myopia’, ‘hyperopia’, ‘astigmatism’, and ‘strabismus’ (in German: “Kurzsichtigkeit”, “Weitsichtigkeit”, “Hornhautverkrümmung” and “Schielen”).

#### Survey of health-related quality of life

Health-related quality of life was assessed using the German version of the KINDL-R questionnaire for parents and children [9–11]. The KINDL-R covers 6 dimensions of quality of life and yields one total scale from 6 subscales (i.e. ‘physical well-being’, ‘emotional well-being’, ‘self-esteem’, ‘family’, ‘friends’, and ‘school’ aspects (in 3 to 6 year-old children in preschool and daycare)). Each subscale contains four items. Each question was to be answered as ‘never’, ‘seldom’, ‘sometimes’, ‘often’, or ‘always’. Computation of scores was performed as described by Ravens-Sieberer et al. [10]. Scores were normalized to a range from 0 to 100, with higher scores indicating better health-related quality of life. Parent-reports were obtained for children between 3 and 17 years of age; self-reports were additionally collected in children between 11 and 17 years of age. Both data sources were analyzed separately. Using the multi-trait analysis program of the New England Medical Center at Tufts University in Boston, the scale fit of the parental-reported KINDL-R survey was 98%. Cronbach’s alpha as measure for internal consistency was α = 0.70 for almost all subscales; for the total score it was α = 0.85 [10]. The values for Cronbach’s alpha of the KINDL-R questionnaire for the subsamples of this study are given in the Additional file 1: Table S1 and Additional file 2: Table S2.

#### Examination of mental health problems

Mental health problems were assessed via the German version of the Strengths and Difficulties Questionnaire (SDQ) for parents [12]. The SDQ covers 4 dimensions of internalizing (‘emotional symptoms’ and ‘peer problems’) and externalizing mental health problems (‘conduct problems’, ‘hyperactivity/inattention’) as well as prosocial behavior. Each subscale contains five items and each item is rated on a 3-point rating scale ranging from ‘not true’ to ‘somewhat true’ to ‘certainly true’. Response categories are assigned arbitrary scores ranging from 0 to 2. Subscale scores are created as the sum of these scores. Hence, subscale scores lead to values between 0 and 10. A total difficulties score is calculated on the subscales of emotional symptoms, peer problems, conduct problems, and hyperactivity/inattention (range 0 to 40). With the exception of prosocial behavior, a higher score generally indicates more mental health problems. Cronbach’s alpha results for the SDQ subscales for the subsamples of this study are displayed in Additional file 3: Table S3. To investigate more clinically relevant mental health problems, subscale scores were also dichotomized as either normal or borderline/abnormal by applying English population-based reference values [13], which have also been used in a population-based sample from Germany [14]. Participants above a certain threshold, i.e. a score in the total scales and subscales, were categorized as children and adolescents with mental health problems. The threshold value for the total score was 13, for ‘emotional symptoms’ 3, for ‘peer problems’ 2, for ‘conduct problems’ 2, and for ‘hyperactivity/inattention’ 5.

#### Definition of potential confounders

Other chronic health conditions, the gender and age of the child, number of siblings, type of daytime care, socio-economic status (parental education, job position, and income) [15], and migrant background [16] had been obtained by parental questionnaires. The socio-economic status of parents was based on income, education, and occupation and was grouped into 3 categories (high, medium, low). The presence of other chronic medical conditions was investigated by using the Children with Special Health Care Needs (CSHCN) Screener [17]. This instrument takes into account whether a child needs or uses medicine prescribed by a physician; needs or uses more medical care, mental health or educational services than is usual for most children of the same age; whether the child is limited or prevented in any way in his/her ability to do the things most children of the same age can do; whether he/she needs or receives special therapy, such as physical, occupational or speech therapy; or has any kind of emotional, behavioral, or developmental problem for which the child needs or receives treatment or counseling.

### Statistical analysis

For the present analysis, all children under 3 years of age were excluded because data on mental health and health-related quality of life were not available for this age group.

Descriptive statistics such as frequencies, percentages, means, and standard errors were used for demographic and clinical variables. Cohen’s d and linear regression analysis were used to evaluate the difference between children with and without strabismus in health-related quality of life (KINDL total scale and subscales) and mental health (SDQ total scale and subscales).

For calculating Cohen’s d with respect to health-related quality of life, the differences in means and standard deviations were taken from the present analysis. For linear regression, crude (unadjusted) and adjusted effect estimates, *p*-values, and 95% confidential intervals were calculated.

#### Association analysis

Effect estimates (betas and odds ratios) were calculated without and with adjustment for the following confounders: age (in years), gender (male/female), presence of siblings (yes/no), socio-economic status (low, medium, high), migrant background (yes/no), place of residence (urban/small town/town/city), East/West Germany, presence of another chronic medical condition (yes/no), type of day care (exclusively within the family yes/no), and officially confirmed disability (a permanent physical or mental disability that is accepted by an evaluation procedure of the maintenance council). Before conducting the analysis, potential confounders were identified by using a causal diagram and directed acyclic graphs (Additional file 8: Figure S8).

If an association between the presence of strabismus and SDQ scales was found, binary logistic regression analysis was used to investigate the strength of the association between the presence of strabismus and a borderline/abnormal classification of the SDQ scales.

Analyses were carried out using sampling weights (defined by age, sex, region, and citizenship) and Complex Samples procedures to achieve a representative sample [18] using the IBM Statistical Package for the Social Sciences (SPSS) version 21.0.

## Results

Of 14,835 children and adolescents at the age of 3 to 17 years, 579/12,989 (4.5%) were reported to have strabismus (*n* = 1522 (10.3%) with missing answer, *n* = 324 (2.2%) with answer “don’t know”). Basic characteristics of the total sample and of children with strabismus are described in Table 1. With respect to health-related quality of life scales and mental health problems, missing data in parental reports were associated with migrant status and living in cities as well as missing data for other covariates (Additional file 4: Table S4). Missings in self-reported health related quality of life were associated with migrant status, the presence of chronic disease, and having an official disability (Additional file 5: Table S5).

**Table 1:** Characteristics of the study sample and the subgroup of children with strabismus. Data from the KiGGS Study 2003–2006

Characteristic, % (n)	KiGGS Study sample *N* = 17,640	Study sample for analysis (3–17 years) *N* = 12,989	Children with strabismus (3–17 years) *N* = 579
Sex: female	48.7% (8655)	48.9% (6346)	51.5% (298)
Age:			
0–2 years	13.6% (2805)	–	–
3–6 years	21.0% (3875)	27.5% (3577)	21.9% (127)
7–10 years	21.7% (4148)	28.6% (3714)	28.5% (165)
11–13 years	17.3% (3076)	20.5% (2659)	23.5% (136)
14–17 years	26.3% (3736)	23.4% (3039)	26.1% (151)
Sibling: yes	70.9% (12,506)	76.8% (9975)	75.0% (434)
Day care exclusively			
within the family	22.9% (4037)	14.0% (1824)	10.9% (63)
Residence: rural/small town/ town/city	17.8% / 27.5% / 29.3% / 25.4%	23.0% / 26.3% / 28.8% / 21.9%	22.1% / 29,7% / 30.2% / 18.0%
Migrant: yes	17.1% (2590)	13.2% (1716)	11.1% (64)
Socio-economic status:			
Low	27.2% (4794)	26.8% (3485)	28.5% (165)
medium	45.3% (7997)	46.9% (6093)	46.5% (269)
high	25.1% (4423)	25.7% (3340)	24.3% (141)
Chronic diseases: yes	12.6% (2226)	14.2% (1850)	24.7% (143)
Official disability: yes	2.0% (352)	2.1% (275)	6.7% (39)

In general, children with strabismus had lower scores regarding all the health-related quality of life scales under study (Table 2, Additional file 6: Table S6 and Additional file 7: Table S7). This was found irrespectively of parental-reported or self-reported health-related quality of life. However, relevant differences were only found for the subscale ‘friends’ (parent-reports: 75.3 ± 0.71 [with strabismus] vs. 78.3 ± 0.15 [without strabismus] (*p* < 0.001); self-reports: 74.4 ± 1.02 [with strabismus] vs. 77.7 ± 0.23 [without strabismus] (*p* = 0.002)). For the total scale, Cohen’s d was − 0.15 (parental report), showing a small effect size of strabismus on health-related quality of life. Similarly, Cohen’s d was − 0.18 for the subscale ‘friends’. In simple linear regression analysis of parent-reported scales, strabismus was negatively associated with ‘physical well-being’, ‘emotional well-being’, ‘self-esteem’, and ‘friends’. In analyses adjusted for potential confounders, strabismus remained related to the subscale ‘friends’ (p = 0.002) (Table 3). Regarding self-reports, there were negative associations between strabismus and the subscales ‘family’ and ‘friends’. In adjusted analyses, strabismus remained associated with the subscale ‘friends’ (*p* = 0.003) (Table 3).

**Table 2:** Parent-reported health-related quality of life scores stratified by the presence/absence of strabismus (age 3–17 years). Data from the KiGGS Study 2003–2006

Health-related quality of life domain	No strabismus *N* = 12,245	Strabismus *N* = 567	Cohen’s d	*p*-value
Total scale	77.2 ± 0.12	75.3 ± 0.43	−0.15	< 0.001
Physical well-being	77.8 ± 0.24	75.6 ± 0.82	−0.14	0.007
Emotional well-being	81.2 ± 0.14	79.7 ± 0.59	−0.09	0.016
Self-esteem	70.0 ± 0.16	67.9 ± 0.68	−0.12	0.003
Family	78.5 ± 0.15	77.5 ± 0.57	−0.06	0.08
Friends	78.3 ± 0.15	75.3 ± 0.71	−0.18	< 0.001
School	77.4 ± 0.19	75.8 ± 0.83	−0.08	0.07

Results are given as mean ± standard error. Statistics were performed by linear regression model for a complex sample structure. *P*-values are only given for descriptive purposes

**Table 3:** Associations of strabismus with health-related quality of life scores. Data from the KiGGS Study 2003–2006

Health-related quality of life domain	Parent-reports (age 3–17 years)		Self-reports (age 11–17 years)	
	Crude Beta *N* = 12,812	Adjusted Beta^a^ *N* = 11,671	Crude Beta *N* = 5589	Adjusted Beta^a^ *N* = 5145
Total scale	−1.88 [−2.77; − 0.99];	− 0.95 [− 1.78; − 0.11];	− 1.67 [−3.03; − 0.31];	−1.30 [− 2.65; 0.06];
Physical well-being	− 2.21 [− 3.81; − 0.60];	−0.70 [− 2.40; 1.01];	−0.50 [− 2.70; 1.69];	0.30 [− 1.89; 2.50];
Emotional well-being	−1.46 [− 2.64; − 0.27];	−0.77 [− 1.95; 0.41];	−0.80 [− 2.49; 0.88];	−0.74 [− 2.42; 0.95];
Self-esteem	−2.10 [− 3.48; − 0.71];	− 1.25 [− 2.72; 0.22];	−1.22 [− 3.65; 1.20];	−0.42 [− 2.85; 2.02];
Family	− 1.02 [− 2.18; 0.13];	− 0.32 [− 1.51; 0.87];	− 2.34 [− 4.38; − 0.30];	− 1.77 [− 3.94; 0.41];
Friends	−2.99 [− 4.41; − 1.57];	−2.16 [− 3.53; − 0.78];	3.24 [− 5.25; − 1.23];	−3.07 [− 5.10; − 1.03];
School	− 1.60 [− 3.33; 0.12];	− 0.36 [− 1.93; 1.22];	− 1.43 [− 4.04; 1.19];	−1.74 [− 4.27; 0.79];

Results are given as crude and adjusted betas (95% confidence intervals) calculated by linear regression analysis. There is a difference in sample size between crude and adjusted analyses due to missings in the adjustment variables. In addition, parent-reports were available from age 3–17 years, while self-reports were only obtained in 11–17 year-old adolescents

^a^ Adjusted for age, sex, siblings, socio-economic status, migrant background, residence (rural/small town/town/city), East/West Germany, presence of chronic medical condition, type of day care, and official disability

Mental health problems were more frequent in children and adolescents with strabismus compared to those without (Table 4). In linear regression analyses, associations were found between strabismus on the one hand and the total difficulties score on the other hand. Conducting this analysis with the subscales ‘emotional symptoms’, ‘hyperactivity/inattention’, and ‘peer problems’ led to positive associations with strabismus, whereas none were found for the subscales ‘conduct problems’ and ‘prosocial behavior’ (Table 5). These associations were still present after adjusting for potential confounders (Table 5). Finally, logistic regression analyses, adjusted for potential confounders, revealed that strabismus was associated with the total difficulties score (*p* < 0.001) and the subscales ‘hyperactivity/inattention’ (*p* = 0.001) and ‘peer problems’ (p = 0.001), but not ‘emotional symptoms’ (Table 6).

**Table 4:** Parent-reported mental health scores stratified by the presence/absence of strabismus (age 3–17 years). Data from the KiGGS Study 2003–2006

Mental health domain	No Strabismus *N* = 12,320	Strabismus *N* = 575	Cohen’s d	*p*-value
Total score	8.08 ± 0.05	9.39 ± 0.27	0.22	< 0.001
Emotional symptoms	1.73 ± 0.02	2.13 ± 0.09	0.19	< 0.001
Conduct problems	1.94 ± 0.02	2.02 ± 0.07	0.05	0.24
Hyperactivity/inattention	3.03 ± 0.02	3.48 ± 0.13	0.17	0.001
Peer problems	1.40 ± 0.02	1.76 ± 0.09	0.18	< 0.001
Prosocial behavior	7.80 ± 0.02	7.78 ± 0.08	−0.01	0.73

Results are given as mean ± standard error. Statistics were performed by linear regression model for a complex sample structure. *P*-values are given for descriptive purposes only

**Table 5:** Associations of strabismus with parent-reported mental health scores (age 3–17 years). Data from the KiGGS Study 2003–2006

Mental health domain	Crude Beta *N* = 12,895	Adjusted Beta^a^ *N* = 11,708
Total score	1.31 [0.78; 1.84]	1.00 [0.51; 1.49]
Emotional symptoms	0.40 [0.23; 0.57]	0.24 [0.07; 0.41]
Conduct problems	0.08 [− 0.06; 0.23]	0.05 [− 0.10; 0.21]
Hyperactivity/inattention	0.46 [0.19; 0.72]	0.42 [0.18; 0.66]
Peer problems	0.36 [0.19; 0.53]	0.28 [0.11; 0.45]
Prosocial behavior	−0.03 [− 0.18; 0.13]	−0.01 [− 0.18; 0.15]

Results are given as crude and adjusted betas (95% confidence intervals) calculated by linear regression analysis

^a^ Adjusted for age, sex, siblings, socio-economic status, migrant background, residence (rural/small town/town/city), East/West Germany, presence of chronic medical condition, type of day care, and official disability

**Table 6:** Associations of strabismus with borderline/abnormal parent-reported mental health scores (age 3–17 years). Data from the KiGGS Study 2003–2006

Mental health domain	Crude OR (*n* = 12,895)	Adjusted OR^a^ (*n* = 11,708)
Total score	1.78 [1.43; 2.23]	1.59 [1.20; 2.10]
Emotional symptoms	1.51 [1.21; 1.88]	1.26 [0.99; 1.60]
Conduct problems	0.99 [0.80; 1.24]	1.00 [0.78; 1.29]
Hyperactivity/inattention	1.64 [1.28; 2.10]	1.52 [1.15; 2.01]
Peer problems	1.44 [1.15; 1.80]	1.35 [1.05; 1.73]
Prosocial behavior	0.67 [0.13; 3.37]	0.40 [0.08; 1.93]

Results are given as crude and adjusted odds ratios (95% confidence intervals) calculated by binary logistic regression analysis

^a^ Adjusted for age, sex, siblings, socio-economic status, migrant background, residence (rural/small town/town/city), East/West Germany, presence of chronic medical condition, type of day care, and official disability

## Discussion

Although health-related quality of life is nowadays widely measured in ‘adult’ ophthalmology, the impact of ophthalmological diseases on this health domain is rarely investigated in children and adolescents. Based on a representative sample, we analyzed health-related quality of life in children and adolescents with parental-reported strabismus compared to children without strabismus. While previous studies focused on preschool children or evaluated health-related quality of life in case-control studies, we have been able to confirm earlier findings of an association between strabismus and lower health-related quality of life in a population-based sample of individuals aged 3 to 17 years.

In our study, we found out that lower scores in global health-related quality of life were mainly caused by lower values of the subscale ‘friends’, describing the social contact of a child within his or her peer group. Nevertheless, the effect of strabismus on health-related quality of life was rather small (Cohen’s d = − 0.15) when compared to the effect of pain within the last 3 months (d = − 0.40) or special health care needs (d = − 0.58) [19]. In general, the effect sizes were higher in parental-reports of health-related quality of life than in self-reports at age 11 to 17 years (total scale, family, friends), while ‘emotional well-being’ and ‘self-esteem’ only showed a difference in parental-reports, but not in self-reports. This may implicate that parents should be informed that adolescents with strabismus do not perceive an impact in ‘self-esteem’ or ‘emotional well-being’ due to strabismus, but this is either projected by the corresponding parents or incorrectly identified by them as such. Nevertheless, self-reported health-related quality of life with respect to ‘family’ and ‘friends’ is impaired in children and adolescents with strabismus.

Similar to our results of lower values in the subscale ‘friends’, a recent study reported that children older than 6 years would be less likely to invite a twin with a visible squint to a birthday party rather than the twin with orthotropic eyes [4]. Moreover, the multi-ethnic Pediatric Eye Disease Study (MEPEDS), a population-based study in the United States, reported that strabismus was associated with worse general health-related quality of life in preschool children [5]. Using the Pediatric Quality of Life Inventory, the authors found strabismus related to lower scores in the subscales ‘physical health’, ‘psychosocial health’, ‘emotional functioning’, and ‘school functioning’ [5]. In accordance with this study, we also found lower scores of the corresponding parent-reported KINDL subscales in children with strabismus. However, after adjusting for potential confounders, only the subscale ‘friends’ remained as associated with strabismus. This may be due to different age distributions in the two studies: while we analyzed an age range from 3 to 17 years, the MEPEDS investigated preschool children with an age of 25 to 72 months when analyzing general health-related quality of life. In addition, the perception of the vis-à-vis may be different in the two age groups. While smaller children may not feel disturbed by someone fixating with only one eye, older children may recognize the none-fixating eye of the opposite. This may lead to less eye contact and a different social interaction.

This leads to the question as to whether vision-related quality of life could be improved by interventions. Chai et al. reported that children with strabismus compared to those without have lower scores in the National Eye Institute Visual Function Questionnaire-25 [20]. However, the scores *increased* following surgery for strabismus. It was reported that strabismus surgery in patients with childhood-onset strabismus not only restores ocular alignment, but also has significant positive effects on self-esteem and self-confidence [21]. Interestingly, we could not find differences in ‘self-esteem’ and ‘emotional well-being’ in self-reports of children aged 11 to 17 years. This is in contrast to the results of Nelson et al., who analyzed self-reports at older age [21].

In addition to associations with quality of life, we also found that children and adolescents with strabismus were more likely to have impairments in mental health like ‘hyperactivity/inattention’, and ‘peer problems’ in comparison to children without strabismus. Again, these effects remained after adjusting for potential confounders such as the socio-economic status of the parents. Nevertheless, as we did find differences between self-reports and parental reports of health-related quality of life, other aspects like the parents’ behavior (i.e. overprotection) may play an important role. The association between strabismus and mental health is known from other studies [22, 23] as well. Children suffering from infantile esotropia (convergent strabismus) are reported to have increased odds (2.6 times) of developing mental illness compared to healthy controls [22]. This also applies to children with exodeviations (divergent strabismus) [24]. In the later study, a genetic causal link between strabismus and schizophrenia was discussed [24], which may show that genetic background may be considered as one potential explanation for the associations observed. Both, strabismus and mental health impairment might be caused by one common developmental disorder of the central nervous system. This is supported by the finding that both diseases are more common in children who were born preterm [25, 26].

Notwithstanding these considerations, the psycho-social burden of strabismus could be relevant for the induction of mental health impairments [27]. For exotropia and esotropia, the miss-alignment may lead to disturbed interactions with the peer group (as indicated by our results concerning the health-related quality of life subscale ‘friends’), psycho-social stress by bullying, and ultimately impairments in mental health. For psychosocial domains, some evidence for the beneficial effects of strabismus surgery has been reported. Archer et al. used parental proxies to provide measures of children’s responses to strabismus surgery and found better scorings in domains like social relations, resistance/susceptibility, anxiety, and depression [28]. Chai et al. moved one step further and categorized children with strabismus into having heterophoria (latent strabismus i.e. tendency to squint when binocular fusion is interrupted) or heterotropia (manifest strabismus) and compared them to controls. They found for both conditions that children were more likely to be anxious and depressed compared to controls [20]. Lin et al. reported that Chinese children with strabismus have a higher prevalence of markers for emotional problems (i.e. depression and anxiety) and a higher prevalence of life-time alcohol consumption [6]. All these observations and our results support attempts for early identification and treatment of strabismus in childhood, but, as clinical experience shows, restoration of alignment will not be achieved in all individuals and some may later develop manifest strabismus.

### Limitations

There are several limitations of our study. First, we did not examine eye position, but identified strabismus by parental reports. Hence, small but relevant miss-alignments of both eyes might not have been recognized by parents. Nevertheless, most of the children had taken part in the mandatory regular pediatric screening program carried out by pediatricians. For participants of the KiGGS study, the participation rate of this screen program was between 94% (“U6” at 10–12 months of age) and 85% (“U9” at the age of 6 years) [29]. This program includes investigations about the parallel position of both eyes from the age of 6 months to the age of 6 years.

Second, the presence of strabismus was surveyed as life-time occurrence, and we could not account for strabismus surgery in the past. Thus, we cannot rule out that some children in the strabismus group may actually have no longer had any evident strabismus at the time of study. This misclassification might have introduced some bias leading to an under- or overestimation of effects. In addition, our results are based on parental reports of strabismus that were not validated against physician diagnoses.

Third, the impact of visual acuity on health-related quality of life and mental health could not be determined in the present study. Measurements of visual acuity had not been accurately performed across all 167 study sites and, consequently, data have not been released for public use by the Robert Koch Institute.

Fourth, as occlusion therapy was not surveyed, we were not able to incorporate prior ocular occlusion therapy in our model, which may have influenced the occurrence of mental-health problems. In addition, amblyopia and missing stereoscopic vision may have had impact on daily life activities despite the availability of compensations such as spatial vision by shadow images and distance-dependent object sizes. This will mainly impact sport activities such as table tennis, etc.

## Conclusion

In summary, we found impaired health-related quality of life and mental health in children and adolescents with parental report of strabismus. Impairments in health-related quality of life were mainly due to decreased scores in the domains ‘family’ and ‘friends’, describing the quantity and quality of social contacts within a peer group and having small effect sizes. As most of these interactions take place at school, explanations to teachers and better integration into social structures may lead to normalized health-related quality of life in these domains. In addition, children with strabismus were more likely to have increased SDQ scores for ‘emotional symptoms’, ‘hyperactivity/inattention’, and ‘peer problems’. Assuming causal relationships, these findings underscore the needs for further research in the area of early identification and treatment of strabismus in childhood. Clinically, identifying and addressing mental comorbidities in patients with strabismus may further lead to an increase in health-related quality of life later in childhood.

## Additional file

Additional file 1: **Table S1.**Cronbach’s alpha of parent-reported health-related quality of life scores (*N* = 12,989). Data from the KiGGS Study 2003–2006. (DOCX 15 kb)

Additional file 2: **Table S2.** Cronbach’s alpha of self-reported health-related quality of life scores (*N* = 12,989). Data from the KiGGS Study 2003–2006. (DOCX 15 kb)

Additional file 3: **Table S3.** Cronbach’s alpha of parent-reported mental health scores (*N* = 12,989). Data from the KiGGS Study 2003–2006. (DOCX 15 kb)

Additional file 4: **Table S4.** Non-responder analysis for outcome data. Data from the KiGGS Study 2003–2006. NA indicating missing value. (DOCX 17 kb)

Additional file 5: **Table S5.** Non-responder analysis for self-reported KINDL-R outcome data. Data from the KiGGS Study 2003–2006. NA indicating missing value. (DOCX 16 kb)

Additional file 6: **Table S6.** Parental-report of health-related quality of life scores stratified by the presence/absence of strabismus (age 11–17 years). Data from the KiGGS Study 2003–2006. (DOCX 15 kb)

Additional file 7: **Table S7.** Self-reported health-related quality of life scores stratified by the presence/absence of strabismus (age 11–17 years). Data from the KiGGS Study 2003–2006. (DOCX 15 kb)

Additional file 8: **Figure S8.** Directed acyclic graph for identification of potential confounders for health-related quality of life and strabismus. (DOCX 70 kb)

## Abbreviations

95%-CI: 95% confidence interval
CSHCN: Children with Special Health Care Needs
KiGGS: German Health Interview and Examination Survey for Children and Adolescents
MEPEDS: Multi-ethnic Pediatric Eye Disease Study
SDQ: Strengths and Difficulties Questionnaire
