# Supplementary material for: Health-related quality of life and mental health in children and adolescents with strabismus – results of the representative population-based survey KiGGS
Source: Health Qual Life Outcomes. 2019 May 7;17:81. doi: 10.1186/s12955-019-1144-7 (PMC6505127; doi:10.1186/s12955-019-1144-7)
Supplement: Supplementary file 1 — Table S1.Cronbach’s alpha of parent-reported health-related quality of life scores (N = 12,989). Data from the KiGGS Study 2003–2006. (DOCX 15 kb) [file 12955_2019_1144_MOESM1_ESM.docx]

**Additional file 1**

**Table S1.** Cronbach`s alpha of parent-reported health-related quality of life scores (N=12,989). Data from the KiGGS Study 2003-2006.

| **Health-related quality of life domain (KINDL-R)** | **No strabismus**  N= 12,245 | **Strabismus**  N= 567 |
| --- | --- | --- |
| Total scale (3-6 years)*  Total scale (7-17 years)  Physical well-being  Emotional well-being  Self-esteem  Family  Friends  School (3-6 years)*  School (7-17 years) | 0.60  0.68  0.68  0.60  0.65  0.70  0.63  0.63  0.64 | 0.67  0.68  0.69  0.64  0.67  0.72  0.71  0.60  0.67 |

* Cronbach`s alpha for the parent-reported scale ‘school’ must be regarded separately for the indicated age groups as items and item formulations differ
