# Supplementary material for: Health-related quality of life and mental health in children and adolescents with strabismus – results of the representative population-based survey KiGGS
Source: Health Qual Life Outcomes. 2019 May 7;17:81. doi: 10.1186/s12955-019-1144-7 (PMC6505127; doi:10.1186/s12955-019-1144-7)
Supplement: Supplementary file 2 — Table S2. Cronbach’s alpha of self-reported health-related quality of life scores (N = 12,989). Data from the KiGGS Study 2003–2006. (DOCX 15 kb) [file 12955_2019_1144_MOESM2_ESM.docx]

**Additional file 2**

**Table S2.** Cronbach`s alpha of self-reported health-related quality of life scores (N=12,989). Data from the KiGGS Study 2003-2006.

| **Health-related quality of life domain (KINDL-R)** | **No strabismus**  N= 5,317 | **Strabismus**  N= 272 |
| --- | --- | --- |
| Total scale  Physical well-being  Emotional well-being  Self-esteem  Family  Friends  School | 0.65  0.58  0.57  0.68  0.71  0.52  0.53 | 0.67  0.58  0.54  0.63  0.75  0.58  0.53 |
