# Supplementary material for: Health-related quality of life and mental health in children and adolescents with strabismus – results of the representative population-based survey KiGGS
Source: Health Qual Life Outcomes. 2019 May 7;17:81. doi: 10.1186/s12955-019-1144-7 (PMC6505127; doi:10.1186/s12955-019-1144-7)
Supplement: Supplementary file 3 — Table S3. Cronbach’s alpha of parent-reported mental health scores (N = 12,989). Data from the KiGGS Study 2003–2006. (DOCX 15 kb) [file 12955_2019_1144_MOESM3_ESM.docx]

**Additional file 3**

**Table S3.** Cronbach`s alpha of parent-reported mental health scores (N=12,989). Data from the KiGGS Study 2003-2006.

| **Mental health domain**  **(SDQ questionnaire)** | **No Strabismus**  N=12,320 | **Strabismus**  N= 575 |
| --- | --- | --- |
| Total score  Emotional symptoms  Conduct problems  Hyperactivity/inattention  Peer problems  Prosocial behavior | 0.69  0.64  0.77  0.54  0.58  0.64 | 0.75  0.71  0.81  0.57  0.65  0.68 |
