# Supplementary material for: Health-related quality of life and mental health in children and adolescents with strabismus – results of the representative population-based survey KiGGS
Source: Health Qual Life Outcomes. 2019 May 7;17:81. doi: 10.1186/s12955-019-1144-7 (PMC6505127; doi:10.1186/s12955-019-1144-7)
Supplement: Supplementary file 4 — Table S4. Non-responder analysis for outcome data. Data from the KiGGS Study 2003–2006. NA indicating missing value. (DOCX 17 kb) [file 12955_2019_1144_MOESM4_ESM.docx]

**Additional file 4**

**Table S4.** Non-responder analysis for outcome data. Data from the KiGGS Study 2003-2006. NA indicating missing value.

| **Characteristic** | **Study sample for analysis**  **(3-17 years)** N=14,835 | Children with missing total KINDL-R scores (parental)  (3-17 years)  N=544 | Children with missing total SDQ score (parental)  (3-17 years) N=577 |
| --- | --- | --- | --- |
| Sex (female) | 49.0% (7266) | 46.3% (252) | 48.9% (282) |
| Age:  3-6 years  7-10 years  11-13 years  14-17 years | 26.1% (3875)  28.0% (4148)  20.7% (3076)  25.2% (3736) | 24.6% (134)  22.1% (120)  20.0% (109)  33.3% (181) | 18.5% (107)  25.5% (147)  18.9% (109)  37.1% (214) |
| Sibling (yes) | 74.9% (11,114)  NA= 831 | 32.7% (178)  NA=331 | 50.4% (291)  NA=240 |
| Day care exclusively within the family | 14.0% (2077)  NA=327 | 15.3% (83)  NA=297 | 13.3% (77)  NA=218 |
| Residence (rural/small town/ town/city) | 22.1% / 26.3% / 28.8% / 22.8% | 10.7% / 19.5% / 32.4% / 37.5% | 14.6% / 23.1% / 30.0% / 32.4% |
| Migrant (yes) | 15.0% (2,229)  NA=62 | 65.8% (358)  NA=26 | 43.8% (253)  NA=25 |
| Socio-economic status:  Low  medium  high | 26.8% (3,976)  45.7% (6,773)  25.0% (3,705)  NA=381 | 26.8% (146)  13.4% (73)  2.9% (16)  NA=309 | 20.6% (119)  22.7% (131)  17.0% (98)  NA= 229 |
| Chronic diseases (yes) | 14.1% (2,098)  NA=1283 | 2.8% (15)  NA=440 | 5.2% (30)  NA=316 |
| Official disability (yes) | 2.2% (330)  NA=334 | 2.9% (16)  NA=294 | 2.4% (14)  NA=217 |
| Strabismus (yes) | 3.9% (579)  NA=1846 | 2.9% (16)  NA=294 | 2.4% (14)  NA=217 |
