# Supplementary material for: Health-related quality of life and mental health in children and adolescents with strabismus – results of the representative population-based survey KiGGS
Source: Health Qual Life Outcomes. 2019 May 7;17:81. doi: 10.1186/s12955-019-1144-7 (PMC6505127; doi:10.1186/s12955-019-1144-7)
Supplement: Supplementary file 5 — Table S5. Non-responder analysis for self-reported KINDL-R outcome data. Data from the KiGGS Study 2003–2006. NA indicating missing value. (DOCX 16 kb) [file 12955_2019_1144_MOESM5_ESM.docx]

**Additional file 5**

**Table S5.** Non-responder analysis for self-reported KINDL-R outcome data. Data from the KiGGS Study 2003-2006. NA indicating missing value.

| **Characteristic, %(n)** | **Study sample for analysis**  **(11-17 years)** N=6,812 | Children with missing total KINDL-R scores (self-report)  (11-17 years)  N=164 |
| --- | --- | --- |
| Sex (female) | 48.7% (3,320) | 40.9% (67) |
| Age:  11-13 years  14-17 years | 45.2% (3,079)  54.8% (3,736) | 48.2% (79)  51.8% (85) |
| Sibling (yes) | 73.6% (5,016) | 61.6% (101)  NA=28 |
| Day care exclusively within the family | 13.7% (936)  NA=194 | 15.2% (25)  NA=14 |
| Residence (rural/small town/ town/city) | 21.9% / 26.0% / 28.7% / 23.4% | 21.3% / 23.8% / 28.0% / 26.8% |
| Migrant (yes) | 15.5% (1,054)  NA=4 | 65.8% (358)  NA=26 |
| Socio-economic status:  Low  medium  high | 26.1% (1,777)  46.8% (3,191)  23.6% (1,609)  NA=235 | 34.8% (57)  40.9% (67)  11.6% (19)  NA=21 |
| Chronic diseases (yes) | 15.4% (1,048)  NA=540 | 32.9% (54)  NA=38 |
| Official disability (yes) | 2.6% (179)  NA=205 | 23.8% (39)  NA=15 |
| Strabismus (yes) | 4.2% (287)  NA=1,114 | 23.8% (39)  NA=15 |
