# Supplementary material for: Health-related quality of life and mental health in children and adolescents with strabismus – results of the representative population-based survey KiGGS
Source: Health Qual Life Outcomes. 2019 May 7;17:81. doi: 10.1186/s12955-019-1144-7 (PMC6505127; doi:10.1186/s12955-019-1144-7)
Supplement: Supplementary file 6 — Table S6. Parental-report of health-related quality of life scores stratified by the presence/absence of strabismus (age 11–17 years). Data from the KiGGS Study 2003–2006. (DOCX 15 kb) [file 12955_2019_1144_MOESM6_ESM.docx]

**Additional file 6**

**Table S6.** Parental-report of health-related quality of life scores stratified by the presence/absence of strabismus (age 11-17 years). Data from the KiGGS Study 2003-2006.

| **Health-related quality of life domain** | **No strabismus**  (n= 5,347) | **Strabismus**  (n= 282) | Cohen’s d | p-value |
| --- | --- | --- | --- | --- |
| Total scale  Physical well-being  Emotional well-being  Self-esteem  Family  Friends  School | 74.5±0.18  74.6±0.29  79.4±0.21  67.5±0.26  76.5±0.24  77.3±0.21  71.6±0.29 | 72.0±0.66  72.8±1.17  76.9±0.91  65.3±1.05  74.2±0.92  72.7±1.07  69.8±1.14 | -0.20  -0.09  -0.16  -0.12  -0.13  -0.30  -0.09 | <0.001  0.12  0.006  0.048  0.017  <0.001  0.14 |

Results are given as mean ± standard error. Statistics were performed by linear regression model for a complex sample structure. P-values are given for descriptive purposes only.
