# Supplementary material for: Health-related quality of life and mental health in children and adolescents with strabismus – results of the representative population-based survey KiGGS
Source: Health Qual Life Outcomes. 2019 May 7;17:81. doi: 10.1186/s12955-019-1144-7 (PMC6505127; doi:10.1186/s12955-019-1144-7)
Supplement: Supplementary file 7 — Table S7. Self-reported health-related quality of life scores stratified by the presence/absence of strabismus (age 11–17 years). Data from the KiGGS Study 2003–2006. (DOCX 15 kb) [file 12955_2019_1144_MOESM7_ESM.docx]

**Additional file 7**

**Table S7.** Self-reported health-related quality of life scores stratified by the presence/absence of strabismus (age 11-17 years). Data from the KiGGS Study 2003-2006.

| **Health-related quality of life domain** | **No strabismus**  (n= 5,317) | **Strabismus**  (n= 272) | Cohen’s d | p-value |
| --- | --- | --- | --- | --- |
| Total scale  Physical well-being  Emotional well-being  Self-esteem  Family  Friends  School | 72.9±0.16  70.9±0.27  81.3±0.21  58.4±0.26  82.2±0.23  77.7±0.23  66.6±0.32 | 71.2±0.69  70.4±1.05  80.5±0.82  57.2±1.18  79.9±1.00  74.4±1.02  65.2±1.34 | -0.14  -0.03  -0.05  -0.06  -0.14  -0.20  -0.06 | 0.016  0.65  0.35  0.32  0.025  0.002  0.28 |

Results are given as mean ± standard error. Statistics were performed by linear regression model for a complex sample structure. P-values are given for descriptive purposes only.
