# Supplementary material for: Health-related quality of life and mental health in children and adolescents with strabismus – results of the representative population-based survey KiGGS
Source: Health Qual Life Outcomes. 2019 May 7;17:81. doi: 10.1186/s12955-019-1144-7 (PMC6505127; doi:10.1186/s12955-019-1144-7)

**Additional file 8**

**Figure S8.** Directed acyclic graph for identification of potential confounders for health-related quality of life and strabismus.


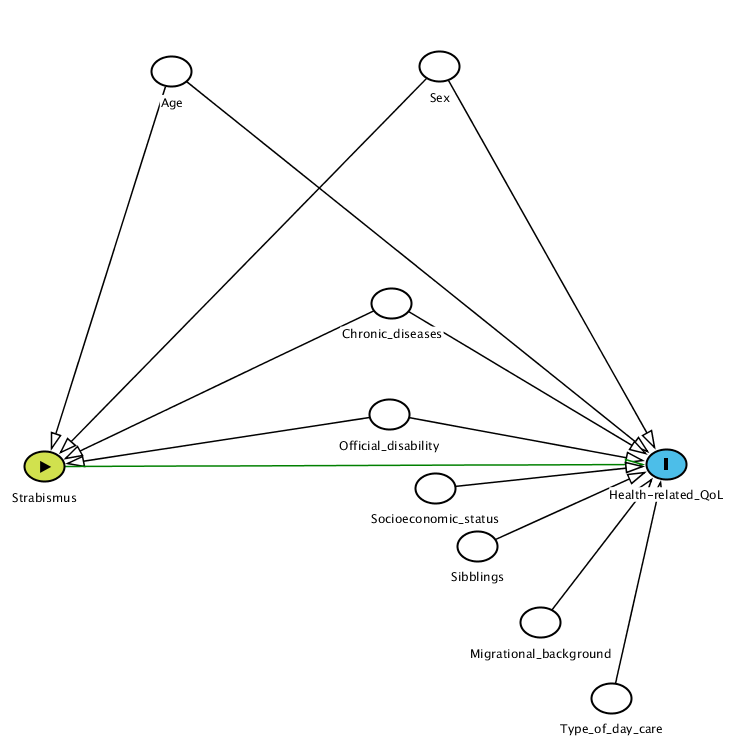

Supplement: Supplementary file 8 — Figure S8. Directed acyclic graph for identification of potential confounders for health-related quality of life and strabismus. (DOCX 70 kb) [file 12955_2019_1144_MOESM8_ESM.docx]
